# Temporality in the social sciences: New directions for a political sociology of time

**DOI:** 10.1111/1468-4446.12938

**Published:** 2022-03-24

**Authors:** Birgan Gokmenoglu

**Affiliations:** ^1^ Department of Sociology London School of Economics and Political Science London UK

**Keywords:** historicity, political time, power and resistance, sociology of the future, sociology of time, time and politics

## Abstract

Time and temporality are common themes in the social sciences and sociology. The sociological literature on time remains solipsistically empirical, while theoretical elaborations are focused on modernity, capitalism, and technology, through notions of speed and acceleration. Although existing studies on time are imbued with political issues and processes, as the subfield that studies relations of power and politics, political sociology has yet to consolidate a temporal lexicon for studying structures of power and political phenomena. This review situates three recent books on time and politics within a broader sociological literature on time and calls for a political sociology of time. I argue that developing a conceptual apparatus that takes time as an element of power is fundamental to building dialogue across the empirical material and across disciplines. I conclude by offering three avenues for the development of a political sociology of time.

The Political Value of Time: Citizenship, Duration, and Democratic Justice. E. F. Cohen . Cambridge University Press, 2018. 183 pp. £18.00 (paperback).

Time and Power: Visions of History in German Politics, from the Thirty Years’ War to the Third Reich. C. Clark . Princeton University Press, 2019. 312 pp. £25.00 (hardcover) £14.99 (paperback).

Familiar Futures: Time, Selfhood, and Sovereignty in Iraq. S. Pursley . Stanford University Press, 2019. 320 pp. £22.00 (paperback).

*The Political Value of Time: Citizenship, Duration, and Democratic Justice*. E. F. Cohen. Cambridge University Press, 2018. 183 pp. £18.00 (paperback).

*Time and Power: Visions of History in German Politics, from the Thirty Years’ War to the Third Reich*. C. Clark. Princeton University Press, 2019. 312 pp. £25.00 (hardcover) £14.99 (paperback).

*Familiar Futures: Time, Selfhood, and Sovereignty in Iraq*. S. Pursley. Stanford University Press, 2019. 320 pp. £22.00 (paperback).

Time is as political as it is social. It is social because our understanding of the directionality of time, its measurement, allocation, and overall meaning is historically and geographically specific. That is to say, if we take advanced industrial countries of the West as an example, that time was not always understood as a linear and sequential progression of days, months, and years; it was not always measured by clocks and calendars; it was not always allocated according to the distinction between “work” and “life;” and time was not always money. Instead, these temporal structures and norms are the result of specific historical processes in specific contexts, like the transition to industrial capitalism or modernity and post‐modernity. The temporal order of our lives is constructed and maintained by practices and structures of thought that only make sense as long as those practices and logics are collectively or inter‐subjectively produced and reproduced. Time is political to the extent that those practices and logics are contested, negotiated, and reconfigured.

Indeed, time is intertwined with power relations. Examples abound: French revolutionaries promulgated a new calendar to mark the Revolution's rupture with the past (Perovic, 2012); schedules contribute to the establishment of social hierarchies (Zerubavel, 1985); nation‐building requires the homogenization of time (Anderson, 2006 [1983]); time in industrial capitalism is a “currency” that is exchanged in the labor market (Thompson, 1967); states make certain social groups wait as a form of subjugation (Schwartz, 1974), such as non‐citizens for visas (Parla, 2020), the working class for social services (Auyero, 2012), the unemployed for welfare programs (Ozolina, 2019), and the list can be extended. Even though sociologists have long been thinking about the temporal aspects of political phenomena, as the subfield that studies politics, relations of power, social divisions, and political institutions, processes, and change, political sociology has yet to develop a lexicon for thinking about time.

This lack of a common vocabulary to talk about time in politics has led to the commonly observed fragmentary state of the literature on time and temporality. There is a wealth of article‐length empirical studies on time that talk past one another, using the same word to mean different things. Take, for example, the word “temporality.” While it may denote an individual's experience of time, it may also refer to how the past, the present, and the future are tied together in a particular narrative. This polysemy entails each author defining their terms anew in each study or coining a new term to fit their own cases without necessarily being transferable to others, thus adding to the fragmentary state of the existing literature. Considering the accumulative and dialogic nature of knowledge production and the rich pool of empirical studies we have on time, I suggest that political sociologists begin thinking *conceptually* about time as a dimension of power. I take conceptualization to be a way of categorizing observed patterns in socio‐political reality (Swedberg, 2014) and a step toward systematically incorporating the temporal dimension into our theories about power relations, politics, and social change.

My argument consists of three interrelated stages: First, I develop further the observation that there is an abundance of empirical studies that use time either as an object of inquiry or an explanatory dimension of socio‐political phenomena, but that these studies have yet to develop a conceptual or theoretical common ground, with few exceptions; second, I argue that developing a conceptual toolkit about time as an element of power would help build meaningful dialogue both across the empirical material and across subfields and disciplines; and third, that conceptualization could lead to broader theorization, which would be useful for (a) sociologists and political sociologists to categorize the empirical clutter to better understand the workings of time as an inseparable dimension of social and political reality, and (b) for thinking about time *politically,* by making the familiar unfamiliar, thus exposing injustices and inequalities that are engrained in the complex temporal dynamics of socio‐political life.

In what follows, I will first review the sociological literature on time and temporality. Then, due partly to the telling absence of a recent book‐length publication on time in the discipline of sociology, and partly to the inherent interdisciplinarity of discussions on time, I will turn to neighboring disciplines to review, in greater detail, three books that deal with time and politics that are productive for sociologists to engage with, owing to their rich conceptual offerings. Lastly, drawing on these books and my discussion of them, I will call for a political sociology of time and suggest a few directions that conceptual or theoretical work in this field can take.

## 1 A BRIEF INTRODUCTION TO TIME IN SOCIOLOGY

My intention here is not to provide an exhaustive overview of the literature but to briefly sketch the place of time and temporality in sociology, emphasizing time's prominence across sociological subfields.

From Marx to Durkheim, Simmel to Arendt, Elias to Giddens, social theorists and sociologists have dealt with time in their analyses of the social. Some subfields of sociology have been more reflexive than others about their use of the category of time in their studies. One such subfield is historical sociology, whose treatment of history and events has evolved through its close contact with the discipline of history (e.g., the works of William H. Sewell Jr, Andrew Abbott, and Charles Tilly); another is the sociology of gender, especially in its use of social reproduction theory, where scholars have shown the unequal distribution of time between genders and the inequalities that this gendered distribution of time creates (Bryson, 2007; Fraser, 2000; Weston, 2002). Sociology of work has also been sensitive to time, especially through studies of post‐work, leisure, acceleration, and the temporalities of work (e.g., Hochschild, 1997; Sharma, 2014). Sociologists of technology have been attentive to social time in the relationship between technology and society (e.g., Bijker & Law, 1992; Wajcman, 2004). Memory studies have long offered insights into the socio‐political implications of collective memory, bringing the past into focus (e.g., the works of Maurice Halbwachs; Lowenthal, 1985).

Besides time being a common theme in subfields of sociology, we can also talk of a sociology of time, where time is taken as the object and the focus of inquiry for theorizing the social. Eviatar Zerubavel and Barbara Adam have led this subfield from the late '70s and early '90s onwards, respectively, with contributions such as *Hidden Rhythms* (Zerubavel, 1985), *Time Maps* (Zerubavel, 2003), *Time and Social Theory* (Adam, 1990), and *Timescapes of Modernity* (Adam, 1998), among countless other books and articles. Starting from the late '90s and early 2000s, we see increasing attention among sociologists of time to speed and acceleration (Beckert, 2016; Martineau, 2015; Rosa, 2013; Vostal, 2016; Wajcman & Dodd, 2017), and to the relationship between time and technology, digitalization, and automation (Bauman, 2000; Castells, 1996, 2009; Wajcman, 2015). Overall, the sociology of time has focused on time in capitalism and modernity as the two defining structural features of contemporary societies. Thanks to this scholarship, and others in different disciplines, there is now a temporally oriented theoretical common ground to converse about acceleration, speed, and technology in capitalism and modernity.

Scholarly attention to time has been developing hand in hand with contemporary debates on the ground. For example, in the last two decades, we have witnessed calls for, and the emergence or resurgence of, various “slow” movements (e.g., slow food, slow academia, slow cities), a feminist time movement (Weeks, 2011), movements for a shorter working week (Coote et al., 2020), and Fridays for Future demonstrations. Alongside these developments, a wide array of academic activity around time emerged such as critical time studies, time‐use studies, conferences and networks dedicated to studies of time, academic journals devoted to the question of time in society (e.g., *Time and Society*, founded by Barbara Adam in 1992), and many special issues on time, temporality, and the future in nonspecialized academic journals.

As this brief overview demonstrates, rather than being a niche topic, studies on time have been widespread. However, scholarship on time remains “unconnected,” “noncumulative,” “fragmentary,” and “solipsistic” (Rosa, 2013, p. 1) at the empirical level, where we see innumerable, usually article‐length, output using time or temporality as a major aspect of analysis, that do not speak to one another. The scarcity of dialogue is largely due to the lack of a coherent theoretical or conceptual framework that provides us with a language to talk about similar phenomena within sociology, or across disciplines for that matter. The exception at the theoretical level, as mentioned before, is the scholarship on capitalism and modernity that deals with the temporal aspects of contemporary societies through notions of speed, acceleration, and questions of technology.

Although existing studies on time are imbued with political issues and processes, political sociology, as a subfield that studies systems of power, has yet to consolidate a temporal lexicon for studying political phenomena. Pockets of research on time in political sociology center around waiting as a practice of social ordering (Auyero, 2012; Ozolina, 2019; Schwartz, 1974) and around the temporal dynamics of contentious politics, particularly of protest events and future imaginations (Della Porta, 2016, 2020; Gillan, 2020; Mische, 2009, 2014). This subfield shares with the rest of the discipline an abundance of empirical studies that use time or temporality that usually fall short of building dialogue. It also suffers from a lack of an explicitly temporal approach to political structures at the theoretical level. Even though gender, technology, capitalism and the like are inherently political, temporally oriented theories of explicitly political processes, institutions, and structures do not yet exist.

In this review, I call for a political sociology of time and argue that the conceptual level can be the connecting thread across the empirical material and across disciplines. Concepts enable us to categorize the empirical clutter and consolidate the building blocks of future theoretical attempts at understanding the political structures of societies. A political sociology of time that approaches time politically as an element of power would help us grasp the politicized nature of the temporal structures of the contemporary world, from authoritarianism to democracy, electoral cycles to political crises.

In the next section, I review three books from different fields and disciplines to tease out concepts that are widely used but insufficiently conceptualized, that nevertheless offer pathways for establishing a temporal lexicon for a political sociology of time, and beyond.

## 2 TIME AND POLITICS IN RECENT PUBLICATIONS

Elizabeth F. Cohen's *The Political Value of Time: Citizenship, Duration, and Democratic Justice* examines the role of time in the exercise of power, situating its main disciplinary contributions in political science and political theory. The book is concise, clear in its arguments and categorizations, and it places time squarely into the heart of political processes as a major factor with political value.

From the first page, Cohen explicitly specifies what time refers to: “scientifically measured durational time,” that is, “distinctly linear” and “measured by clocks and calendars” (p. 1), rather than sociohistorical conceptualizations of time. Drawing on the observation that time is inherent in all political procedures and the exercise of power, the guiding questions of the study are listed as “How does durational time come to structure and distribute political power? Why is durational time so frequently inserted into political procedures for granting, denying, and exercising rights? How can we evaluate the normative effects of the ways that states command the time of their citizens?” (p. 3). Time, then, is understood as a “good that is created and governed in the context of the state” (p. 4), and particularly in the democratic state, liberal democracy, or procedural democracy, used interchangeably throughout the book. The aim is to show how durational time is a political good and to explore “how citizens' time is and ought to be treated by the state” (p. 5). The book thus contributes to political science, theories of liberal democracy, and through its normative insights, to public policy scholarship and contemporary theories of social justice.

Calendrical time stands out as fundamental in political processes that carve out the boundaries of nation‐states, populations, and citizenries within a state. Through “the deadline,” Cohen brings temporal boundaries on a par with the much‐studied spatial or territorial boundaries of the state. Three different deadlines are identified and elaborated, from the least democratic to the most democratic temporal boundary design: fixed single‐date deadlines that mark, for example, the establishment of a nation‐state; countdown deadlines such as temporary visas or work permits that expire at some point in time; and recurring or repeating deadlines such as elections in procedural democracies.

Writing about the democratic potential of the durations that pass between repeating deadlines, Cohen discusses ancient and modern political philosophers, going at length into the 18th‐century mathematician Condorcet's “science of democracy” (p. 81). The discussion around the pitfalls of a “hasty democracy” and Condorcet's institutional prescriptions for a “slow democracy” is particularly relevant for thinking about democracy in today's accelerated society. That said, more contemporary theories of democracy and alternative theories of democracy (other than procedural and representational democracy) are conspicuously missing from the discussion. The exclusive focus on democracy also weakens the book's relevance even for the “established” democracies of our times, as we increasingly witness those democracies backslide into right‐wing populism or a form of authoritarianism.

The book explains the political value of time through the political economy of time. Cohen lists five reasons for why time is an inextricable part of liberal democracy, other than politics taking place within time: its situatedness, quantifiability, objectivity and impartiality, egalitarianism, and its ability to simultaneously appear both situated and objective. These characteristics of time allow it to have instrumental and representational value, in that durations are essential for democratic processes, and time also comes to act as a proxy for those processes such as deliberation or maturation that have political value in democracies. Here, time takes on the role of a unit of commensuration. Cohen likens this “political economy of time” to how for Marx, the amount of time that workers spend in production becomes the measure by which they receive payment. For Cohen, time in politics serves a similar function. By approximating processes required for political procedures, durational time grants or denies certain rights to certain people. Time's central role in politics leads Cohen to discuss temporal injustices and the normative insights gained by this study.

*The Political Value of Time* is a welcome contribution and a good prompt for thinking sociologically about time in its specifically political dimensions. The narrow focus on scientifically measured durational time is both a strength and a limitation. It is a strength because it offers a clear‐cut definition of time that is indisputably observable and relevant, as nothing exists outside of time, and with time's passage, things change and new things happen. However, the book falls short of developing a concept of “political time” (p. 3) due to the limitations of durational time. Political time is treated as “the actual dates and quantities of time used by the state in its capacity as sovereign” in a footnote on page three, and no further conceptualization is made throughout the rest of the book. Concentrating exclusively on duration has left aside more sociological questions of how time becomes a terrain of politics itself, and how it is not only political by virtue of being implicated in political processes but how it is *politicized* by different forces in society and the political domain. The author, in their discussion of value and valuation, has left social relations and relationality outside of the “political economy of time,” while touching on temporal injustices that such a political economy engenders.

Cohen's examination can be a solid starting point for sociologists interested in inequalities and particularly in inequalities emerging predominantly from the narrowly defined political sphere. Sociology is well‐equipped to expand the concept of “the political economy of time” by incorporating analyses of the socio‐political processes through which the politicization, valuation, and hierarchization of time occur in a specific historical context. Such an expansion would bring in the element of contestation into our understanding of time's role in politics, adding to these multidisciplinary efforts toward a conceptualization of political time.

Another implication that this book has for the discipline of sociology is the necessity of attending to time as a medium for the exercise of power, which lands us in political sociology. In *The Political Value of Time,* the sole entity that is responsible for exercising power appears as the state. Although never explicitly defined in the book, the state is referred to in its coercive, or at least structuring, capacities to impose, control, or command. This treatment of the state as the only actor that commands politics, and the exclusive focus on stability, is restrictive, especially since the book's theoretical limits are bounded by liberal democracy, a system that often witnesses bottom‐up political engagement. That said, bringing the state into the discussion is necessary for scholarly discussions of socio‐political change and democracy. Cohen's insights regarding the state's exercise of power through time can be applied to and expanded in sociological studies of other spheres of power, and of other forces or actors in society, that have similar structuring capabilities. Here, time's instrumental and representational values can be used and built on.

Christopher Clark's *Time and Power: Visions of History in German Politics, from the Thirty Years' War to the Third Reich* is very similar in topic to Cohen's *Political Value of Time*, as they both tackle the question of time in a strictly political sphere, at the state level. Clark, however, adopts a broader conceptualization of time when compared to Cohen's relatively narrow analysis based solely on the quantity of time elapsed. Clark's *Time and Power* is a historical account of the historicity of different political regimes, and specifically, of how those who wield political power understand the connections between the past, the present, and the future. Clark does this by examining four different regimes over the span of four centuries, in different historical periods, organized in chronological order, from Friedrich Wilhelm of Brandenburg‐Prussia in the 17th century through to the National Socialists in the 20th century.

As a work of history, this book is more descriptive than conceptual or theoretical and does not set out with a specific theoretical framework. Instead, Clark reviews and makes references to work on time within historical studies, the transition from traditional to modern temporalities, and studies of how regimes of power intervene in the temporal order through calendars and clocks. Even though *Time and Power* does not theorize the relationship between the two phenomena in the title of the book, it is a temporally oriented analytical contribution to these literatures, as well as an excellent secondary source for comparative and/or historical sociologists working on time and temporality, historicity, futurity, and the like.

Clark makes a distinction between historicity and temporality, both of which are vaguely defined terms in the literature that add to the fragmented state of temporal studies. They follow François Hartog's definition of historicity to “denote a set of assumptions about how the past, the present, and the future are connected,” rather than “a doctrine or theory about the meaning of history” or “a mode of historiographical practice” (p. 1). Temporality is used “to denote a political actor's intuitive sense of the texture of experienced time” that captures “a feeling for the motion of time” (p. 6). The vagueness of these characterizations gives them a flexibility that can be useful for empirically oriented studies aiming to explore imaginaries of history and the future, and more suggestive for others that are more theoretically oriented.

The first regime Clark studies in the book is the composite monarchy of Brandenburg in the aftermath of the Thirty Years' War. Clark characterizes this regime as “the history machine,” where political power is seen as “a time machine, an engine that makes history happen” (p. 64). “The history machine” refers to the state in its “forwards‐moving, innovating, tradition‐breaking power” (p. 86). The state is perceived in interaction with other internal and external powers whose actions were unpredictable, with the Elector as the decision maker who resolves the uncertain, open‐ended future in order to shape it. Acting with this historical mission “meant discarding tradition, apprehending the multiplicity of possible futures, identifying threats posed by each, and selecting among them” (p. 64). The past, in the form of tradition, was increasingly overcome by the impositions of the future, through the trope of necessity, dire need, emergency, and imminent dangers or threats. The elector monopolized the authority to determine when it was necessary to uphold tradition and law, and for how long. Necessity eventually “transformed from an ad hoc argument for provisional interventions of central power,” and was “temporally stretched. It referred less and less to a clear and present danger and more and more to a permanent anticipatory posture, a security apparatus focused on future contingencies” (p. 40). Institutions were built on these permanent anticipatory postures and future contingencies.

*Time and Power* then examines the rule of “the historian king” Frederick II, who earned this title due to his studies of the history of Brandenburg‐Prussia. The king's writings served a propagandist function, “not just for the present […] but for posterity” (p. 79). However, what influenced his understanding of historicity and temporality was his socially conservative leanings and his aristocratic lifestyle. Frederick II aimed to restore the economic stability of the nobility and preserve the privileges of the estate‐owning class, which affected how he wrote history: the “past was brought into conformity with the political and social objectives of the Frederician state” (p. 95). Indeed, Frederick II was quite ahistorical in his view of the state, as he viewed it as an “extra‐historical fact” that encapsulated immutable, universal laws (p. 97). History unfolded cyclically (p. 98), recursively (p. 109), and thus the present and the past were analogical; “time was pleated” (p. 109). The state was a timeless entity, instead of a historical one for Frederick II.

Another temporal rationale for conservatism and statism that justifies the preservation of the power of the state is in Bismarck. Bismarck's rule and his understanding of historicity were marked by the revolutions of 1848 which characterized the period by discontinuity, turbulent politics, and fundamental changes to the state, politics, and history. The statesman, in this radically volatile political terrain, was the “boatman on the river of time,” floating above the waves of transformation by way of the enduring structures of the monarchical state. Bismarck knew that the revolution was irreversible, so he sought continuity in the power of the state, with a willingness to impose authoritarian measures in a crisis (p. 159).

The statism and conservatism of earlier periods, where the survival of the state is synonymous with the motion of history, ends abruptly with the National Socialists. Clark writes about the time of the Nazis as a “re‐patterning of temporality” (p. 195) and “a radical rejection of ‘history’” (p. 208). The state, which was the central organizing principle of politics and history, is replaced by race. To bypass the disappointment of the First World War, the regime reaches down to the remote past, so much so, that “the recent political history of Weimar would become astronomically remote, while the millennial antecedents of the new regime […] came to seem (or supposed to seem) very near” (p. 195). To establish an “ahistorical, racial continuum” (p. 210), the regime collapsed the remote past, the present, and the remote future.

The historicity of regimes urges us to think theoretically about time and power. Clark describes the temporal mechanisms by which particular regimes are formed and changed through imaginaries, discursive acts, and institution‐building, without going into conceptual or theoretical discussions. The book focuses mostly on the historicity of individual powerholders, and hence sometimes falls short of delivering its aim of exploring “the historicity of a number of regimes” (p. 3), as less attention is paid to the constellation of powers that construct a regime than to the views of the rulers. Nevertheless, the book raises questions about the connections between configurations of the past, the present, and the future on the one hand, and the strategic and institutional configurations available to power holders on the other; who holds the power to determine “normal times,” “dire times,” “emergencies,” or other similar temporal characterizations of the defining features of a certain period; and the relationship between the conservative‐liberal spectrum and historicity. Perhaps most importantly for this review, Clark's way of studying historicity is informative for the consolidation of this particular conceptualization in political sociology.

Sara Pursley's *Familiar Futures: Time, Selfhood, and Sovereignty in Iraq* is an unconventional, weighty, and multi‐layered book that defies disciplinary boundaries. It is unconventional in its eclectic methodology, re‐interpretations of historical documents and well‐known events, and its nonlinear and unchronological structure of argumentation. It is weighty because of the multiplicity of research topics with which the book engages while retaining the complexity of social reality and history. Pursley explores “sociohistorical or discursive moments in mid‐20th‐century Iraq in which sexual difference and/or familial life were particularly charged objects of reform in the name of the nation's sovereign and developed future” (pp. 27–28). Major themes are projects and understandings of development, the deployment of temporal and gendered narratives in the exercise of power, and how time itself is understood at different moments in the history of postcolonial Iraq. Thus, the book contributes to the history of Iraq, but also of development and colonialism, as well as political science, sociology, gender studies, and cultural studies through its incisive analyses of nation‐building and nationalism, post‐coloniality, futurity, gender, and revolutions.

The book bursts open the categories of the political and of time. In this book, the political is not confined to the state or state officials only but found within the conjugal family, the education system, agricultural projects, reform initiatives, and more. Pursley allows the category of time to take on multiple meanings and manifest itself in diverse ways, much like Clark does. But unlike *Time and Power*, *Familiar Futures* solidifies different treatments and understandings of time into concepts such as timelessness, revolutionary time, or gendered time that can be transferred outside of the immediate context of the book. I will now turn to a few of those concepts that might contribute to laying out the groundwork of a political sociology of time.

Pursley's premise is that the development projects that were initiated in postcolonial Iraq served to reinforce some of the already existing inequalities through various temporal dynamics. After the revolution of 1958, for instance, the temporal dynamics at play were *jumud*, which means “a frozen state,” or stagnation, in the economy on the one hand, and *tajmid*, which means “freezing,” or suspension, of political mobilization, on the other (p. 154). For the Iraqi state, the end to economic stagnation required political stagnation. Political activity could be “suspended” in the name of “development,” where “the work of expanding the social domain” co‐existed with “the simultaneous deferral of popular sovereignty or democracy into an ever‐receding future” (p. 154).

One way in which the future was made to recede was reproductive futurism. The concept is borrowed from Lee Edelman and refers to the “heteronormative discourse [that] works to defer demands for political change in the present by placing on the ‘tiny shoulders’ of the child the burden of embodying a political future that never arrives” (p. 8). Thinking of reproductive futurism as a hegemonic political imaginary allows Pursley to reveal new dimensions of nationalism and sexual difference. The focus on the forward‐looking, future‐oriented temporality of nationalism shows how the limitless, or timeless, future of development/modernity/capitalism is reinforced in a way that defers demands for change. This deferral is driven also by sexual difference and the conjugal family, which complicates Benedict Anderson's argument on the linear‐historical, homogenous time of the nation. The family, as a stable structural unit, enables the nation's forward trajectory, while children bear the burden of the deferred future. Some women (rural, in the private sphere), associated with biological time and cyclical repetition, become the representation of tradition, stagnation, and a backward trajectory; others (urban, in the social sphere) are seen free of the past, but “trapped in its [the nation's] future” (p. 12). Pursley's attention to futurity not only uncovers new aspects of nationalism, gender, and development, but also provides valuable insights into how futurity can be depoliticizing, gendered, and reproductive of inequalities.

Imaginaries and practices of gendered time are elaborated throughout *Familiar Futures*, and in the modern political imaginary of nationhood, observed in the education system, land settlements, and personal status laws. Pursley's examination of gendered time evades easy summaries, but their analysis of Jawad Salim's *Nusb al‐Hurriyya*, the Monument to Freedom, offers some insight into a gendered time in the nationalist political imaginary of postcolonial, postrevolutionary Iraq. Pursley identifies two temporalities, one that is associated with the universal male subject as the martyr or the revolutionary that operates in a linear‐historical temporality, and one that is associated with femininity in a cyclical temporality. In this imaginary, women's reproductive labor is essential to nationhood, both in the private and public spheres (p. 210). This cyclical temporality is what drives the nation forward into its future, or the revolution, making history along the way (p. 212).

Pursley shows how the rupture that is so commonly associated with revolution is much more complicated than a clear break with the past on a linear, historical trajectory. Instead, Pursley considers the explosive and implosive characteristics of revolutionary time, where the connections between the past and the future are reconfigured, through various political subjects such as the child, the male martyr, and the female political subject in the heterosexual social domain. The familiarity of the futures that open up in the revolution, which gives its name to the title of the book, can be traced back to revolutionary time, gendered time, and reproductive futurism, and their role in the creation of the nation, sexual difference, and political subjectivities.

*Familiar Futures* offers sociologists and especially political sociologists many concepts with which to study time and temporal dynamics, and new ways of conceptualizing old phenomena. The book's insistent attention to futurity and the (de)politicizing work that it does is a much‐needed contribution to the study of time and politics. Pursley adheres to the complexities of social life and history, and to the actors and imaginaries that enable the entanglements of the past and the future. By utilizing an expanded understanding of time and the political, and by incorporating the scholars and thinkers of the region in their interpretations, Pursley manages to take the step from the empirical to the conceptual, making novel (re)conceptualizations.

## 3 CONCLUSION: NEW DIRECTIONS FOR A POLITICAL SOCIOLOGY OF TIME

In reviewing recent books on time and politics, I sought to elicit some of the most current approaches and conceptualizations that exist about the temporal dynamics of political phenomena. The kind of conceptualization that this review calls for requires, following Swedberg (2014), deep knowledge of a case, or preferably, more than one case, so that a meaningful level of abstraction based on empirical reality can be achieved. As I argued earlier in the review, political sociologists have access to a wide pool of empirical studies from which to draw. These studies can be used to compare cases, identify commonalities and differences, come to an understanding of how a particular process works, and reach an explanation that is empirically grounded but generalizable enough to be transferable to similar situations. The variety of terms that are used in the books reviewed in this article, conceptualized to a lesser or greater degree, may be insightful and inspiring entry points for establishing the conceptual backbone of a political sociology of time. However, offering a few or all of these concepts as *the* conceptual toolkit for studying time would be an arbitrary selection dissociated from empirical analysis and the relevant literature. Developing a new concept or a constellation of concepts, on the other hand, exceeds the scope of this review for the same reasons. Instead, based on my discussion of the literature and recent works on time and politics, I will conclude by suggesting a few avenues that the political sociology of time can follow. I will highlight both the theoretical and practical, or political, implications that such temporal new directions in political sociology would entail.

One avenue is toward a temporal theory of political structures based on calendrical time. Much like what Cohen has done in *The Political Value of Time*, this strand of the political sociology of time would study the relationship between the exercise of power and durations, deadlines, or dates. Some questions to be addressed would be: How do states command the time of citizens or noncitizens within their jurisdiction? How do different political regimes, from democracy to authoritarianism, set temporal boundaries for purposes of legitimation, control, or domination? What are the “hidden rhythms” of political life? How is time created and governed outside of the state and other political institutions? How do the challengers of power (e.g., activists, social movements, political parties) utilize time? These and similar questions would take time as a medium for the exercise of power and resistance, posing time as a site of contestation. Theorizing the ways in which time is contested, negotiated, and politicized would not only broaden our theoretical horizon, but could lead to practical lessons for social justice, policy making, and wider socio‐cultural change.

Another fruitful direction is toward understanding political ideologies or attitudes from the prism of how the past, the present, and the future are tied together in a discourse or imaginary. Clark, in *Time and Power,* does this at the level of the individual powerholder. Expanding Clark’s focus from the individual to the social, political sociology can develop ways to understand how ideologies like conservatism, racism, or statism are temporally structured, that is, how each ideology is constructed through the way that the past, the present, and the future are related to one another. Pursley attempts at such an understanding in *Familiar Futures* when examining the discursive hegemonic political project of nationalism, and the different ways of relating the past to the future and back that helped create and maintain that political project. Theorization of ideology and political attitudes would broaden our understanding of political campaigns, the emergence and popularization of new regimes and systems of power, and the role of the relational constellation of the past, the present, and the future in socio‐political change.

A third avenue for political sociologists lies in the study of the role of futurity or the future in politics. The future as a site of politics with consequences for the present has been under‐conceptualized, especially when compared with the extensive literature on the role of the past. Pursley has explored the role of the future through the concepts of stagnation and reproductive futurism, bringing to our attention the depoliticizing work that particular discourses about the future do, especially when they are ascribed to particular political subjects or social groups. This line of theorization would ask questions about who is burdened with the future; how the future is expected to arrive; how uncertainty, hope, or dread affect political action; and both the politicizing and depoliticizing work that imaginaries, discourses, or practices (such as prefigurative action) of the future do. At a time when the future has become such a pressing global problem through issues such as the Anthropocene, sustainability, new economies, or migration, theorizing future orientations and the impact of future expectations on power relations will be increasingly necessary to understand how societies change, how new social divisions form, and how, among whom, and where socio‐political conflict occurs.

These directions are by no means exhaustive, and they can be mixed and matched in different combinations. My aim in this review was not to offer a didactic research agenda but rather, to emphasize the necessity of theorizing time as a dimension of power and politics, to understand time like any other often invisible system of oppression with real consequences. I argued that developing political sociology of time could start from the conceptual level, as sociologists already have access to a wide array of empirical studies on time in politics. Building a political sociological lexicon on time will help build dialogue across subfields and disciplines, and advance our theoretical understanding of how power and resistance work, with practical implications for social movements, social justice, and liberatory politics.
